# Computed tomographic characteristics of craniomandibular osteopathy in 20 dogs

**DOI:** 10.3389/fvets.2024.1436356

**Published:** 2024-09-30

**Authors:** L. A. Pérez López, J. C. Almansa Ruiz, G. Steenkamp, A. Holdsworth

**Affiliations:** ^1^Davies Veterinary Specialists, Hitchin, United Kingdom; ^2^Department of Companion Animal Clinical Studies, Faculty of Veterinary Science, University of Pretoria, Onderstepoort, South Africa; ^3^Bristol Veterinary Specialists, Bristol, United Kingdom

**Keywords:** craniomandibular, tympanic bullae and calvarian osteoproliferation, lion’s jaw, pharyngeal stenosis, external ear canal stenosis

## Abstract

Craniomandibular osteopathy (CMO) is a proliferative, self-limiting, non-neoplastic disease of growing dogs characterised by excessive new bone formation on the skull and mandible. The radiographic findings of CMO are well described; however, limited reports of the computed tomographic (CT) appearance are available. This paper aims to characterise the spectrum of CT findings that can occur with CMO. The study is retrospective, descriptive, multicenter, and includes 20 cases. Age at presentation ranged from 6 weeks to 12 months, with no sex predisposition. Scottish terriers were overrepresented (65%); other breeds included Cairn terrier, Jack Russell terrier, Staffordshire bull terrier, labrador retriever, golden retriever, akita and Slovakian rough-haired pointer (one of each breed). Terrier breeds represented 80% (16/20) of the patient cohort. Mandibular osteoproliferation was present in all patients (marked in 80%, bilateral in 95%), affecting the rostral mandible in 25%, body in 85%, and ramus in 80%. Tympanic bulla osteoproliferation was present in 60% (12/20) of patients (all marked, bilateral in 75%). Cranial osteoproliferation (frontal, parietal, temporal, occipital bones or maxilla, or combinations of them) was present in 90% (18/20) of patients (40% marked, 27% moderate, 33% mild). Nasopharyngeal narrowing was seen in all 12 patients with tympanic bulla osteoproliferation (67% marked, 27% moderate) and caused nearly complete occlusion in two of them. External ear canal stenosis was seen in 55% (11/20) of patients (63% marked, 37% moderate, all bilateral). Temporomandibular joint (TMJ) impingement was suspected in 83% (10/12) of patients with marked tympanic bulla osteoproliferation (75% bilateral). Osteolysis with a moth-eaten pattern was seen in the mandible of 10/20 dogs, the calvarium of 5/20 dogs, and the maxilla of 1/20 dogs (5%). Lymphadenomegaly (mandibular and medial retropharyngeal) was found in 15/20 patients (70% mild, 30% moderate). The most severe CT changes were seen in Scottish terriers. CT allows for detailed characterisation of the bony changes associated with CMO, including the effects occurring secondary to osteoproliferation surrounding the tympanic bullae such as TMJ impingement, external ear canal stenosis, and nasopharyngeal narrowing. Osteoproliferation affecting the cranium and the presence of osteolysis were seen more frequently in this study than previously reported in CMO.

## Introduction

Craniomandibular osteopathy (CMO) is a proliferative, self-limiting, non-neoplastic, developmental disorder of bone that affects growing dogs (1–3). It is most commonly reported in West Highland white terriers (WHWTs) (4) but can affect several other terrier breeds including Scottish (5), Cairn (5), Boston (2), and Airedale terriers (6). It has also been reported in multiple non-terrier breeds, including the: akita (7); boxer (8); Bulgarian shepherd (9); bullmastiff (10–12); Deutsch drahthaar (1); doberman pinscher (13); great dane (2); labrador retriever (2); Pyrenean mountain dog (14); Shetland sheepdog (15); Welsh corgi (16); and crossbreed (17).

CMO most frequently results in bilaterally symmetric new bone proliferation on the mandibles and tympanic bullae (2); however, the cranium, cervical vertebrae, radius and ulna can also be affected (4, 17, 18). Affected dogs usually present between 3 and 12 months of age and there is no known sex predisposition (1). The proliferative new bone formation typically ceases at around 1 year of age, when skeletal maturation is complete (5). The aetiopathogenesis of CMO remains unclear. An autosomal recessive mode of inheritance has been proven in WHWTs (4); however, in other breeds, the mode of inheritance is thought to be more complex than a simple Mendelian model (1). Additionally, it has been postulated that infection with canine distemper virus and *Escherichia coli* could be linked to the development of CMO (19). A disease called Caffey-Silverman Syndrome is described in humans, which shares some similarities with CMO in dogs. Affected patients show cortical bone hyperostosis which predominantly affects the mandibles, scapulae, ribs, clavicles, and long bones, along with swelling of the surrounding soft tissues (20).

The most common clinical signs reported in dogs with CMO are: pain opening the mouth; swelling and pain around the cranium, mandibles, and temporal regions; excessive salivation; lethargy; and anorexia (14, 15, 21). Severely affected patients may struggle or may not be able to open their mouth completely, which can be attributed to the new bone proliferation causing enlargement of the angular process of the mandible and tympanic bullae (5); this can result in a mechanical restriction of jaw motion or further progress to a bony fusion of the temporomandibular joint (TMJ) (2). Neurological signs can develop if calvarial osteoproliferation is severe enough to result in compression of the brain or brainstem (7). CMO can also lead to secondary problems such as chronic otitis (21), exophthalmos (22), and strabismus (22), and has been associated with angular deformity of the carpus (3).

The prognosis for dogs with CMO varies depending on which bones are affected and the severity of the changes. Dogs that develop ankylosis / pseudoankylosis of the TMJ have a poorer prognosis, due to the inability to prehend and masticate food (2).

A diagnosis of CMO is made based on a combination of the signalment, presenting clinical signs, imaging findings, and response to treatment. Historically, the imaging diagnosis has been based on typical radiographic findings, which comprise new bone formation affecting the mandibles, tympanic bullae, and flat bones of the calvarium (including the frontal, temporal, parietal and occipital bones) (10, 12, 15). Despite the increasing use of CT for imaging veterinary patients, to the author’s knowledge, there are only a handful of case reports, a short case series, and a study investigating the mode of inheritance of CMO in Deutsch drahthaar dogs published in the peer-reviewed literature, which include the CT findings seen in patients with CMO (1, 16–18, 21, 23, 24). The CT findings documented to date include periosteal new bone formation affecting the mandibles (1, 16–18, 21, 23, 24), the tympanic bullae (17, 18, 21, 24), and the cranium (17, 18, 23), along with sclerosis affecting the skull bones (16–18, 23). Osteoproliferative changes of varying severity, which predominantly involve smooth benign osseous thickening of the mandible, petrous temporal bone, tympanic bulla and calvarium are also described as CT features of CMO in veterinary radiology textbooks (25–27).

This study aims to describe the spectrum, extent and severity of CT features associated with CMO in dogs.

## Materials and methods

### Selection and description of subjects

This study is a retrospective, multicentre, descriptive case series. The medical records of the Onderstepoort Veterinary Academic Hospital (University of Pretoria) and Davies Veterinary Specialists (Linnaeus) were searched for dogs diagnosed with CMO between January 2011 and December 2023. Owner consent was obtained for all diagnostic tests, treatment, and subsequent use of anonymised clinical data for research purposes. Approval for this study has been granted by the RCVS Ethics Review Panel and the Research Ethics Committee of the University of Pretoria (REC 162–22).

Cases meeting the following criteria were included in the study: (1) signalment, presenting complaint, and physical examination findings were recorded in the clinical notes; (2) a complete CT of the head was performed, and the images were available for review; and (3) the patient was diagnosed with CMO during the referral visit. The diagnosis of CMO was reached by consideration of the signalment, history, physical examination findings, and compatible CT findings.

The database search yielded a total of 20 dogs meeting the inclusion criteria; 13 from the University of Pretoria and 7 from Davies Veterinary Specialists. Signalment, vaccination status, presenting complaint, physical examination findings, treatment and follow-up/outcome (where available) were recorded. The CT images were also obtained and anonymised to remove any direct patient identifiers.

### Data recording and analysis

All CT images from the University of Pretoria were acquired using a Siemens Emotion Duo scanner (Siemens Medical Systems, Forchheim, Germany). CT images from Davies Veterinary Specialists were acquired with a GE High-Speed Dual Slice scanner (GE Hangwei Medical Systems Co Ltd., Beijing, China) for one patient, and a Siemens Somatom Go.All 64-slice scanner (Siemens Medical Systems, Forchheim, Germany) for six patients. The Siemens Emotion Duo and GE High-Speed Dual Slice machines used an axial scanning mode; the Siemens Somatom Go.All 64-slice machine used a helical scanning mode. Scanning parameters varied between machines and patients: 66–225 mAs and 120–130 kV. Collimation (32 × 0.7 mm), pitch (0.55–0.625), and rotation time (1 s) were only retrievable from the metadata of scans acquired using the Siemens Somatom Go.All 64-slice machine. A 600mgI/kg dose of iohexol (Omnipaque TM 300 mg I/ml GE Healthcare) was administered to the six patients scanned with the 64-slice CT machine; the contrast was delivered intravenously at a rate of 2 mL/s using a power injector (CT Motion TM, Ulrich Medical, Germany). The same dose was administered by manual injection to the patient scanned with the GE dual slice scanner. A high spatial frequency bone reconstruction was available for all cases; a medium spatial frequency soft tissue reconstruction was only available for 12/20 cases (including all cases where contrast was administered). The reconstructed slice thickness (0.5–3 mm), slice overlap (0–1.5 mm), and reconstructed FOV (11.1–25.1 cm) applied were variable between patients. CT images were displayed using standard bone and soft tissue windows.

All CT images were reviewed by a second-year ECVDI resident (LP) and an ECVDI-certified radiologist (AH). Image review was performed at a dedicated viewing station using commercially available DICOM viewing software (Osirix MD v.12.0.2, Pixmeo SARL, Bernex, Switzerland). The reviewers evaluated and graded the images separately; a consensus agreement was subsequently reached between reviewers. The reviewers were not blinded to the diagnosis of CMO at the time of image evaluation.

The CT studies were evaluated for evidence of periosteal new bone formation, osteolysis and/or sclerosis affecting the skull. For each part of the skull (e.g., mandible, tympanic bulla, maxilla, calvarium), the severity of any osteoproliferation and/or osteolysis was categorised as absent, mild, moderate or marked. The site (body, ramus, or both) of any osteoproliferative changes affecting the mandible was recorded. Due to the severity of the mandibular changes in multiple patients, it was often impossible to differentiate the periosteal new bone from the underlying cortical bone, or the medullary bone from the cortex. To quantify the severity of the osteoproliferative changes, the mediolateral thickness of the mandible was measured (including any periosteal new bone along the medial and lateral surfaces of the mandible) at the most affected site. The changes were subjectively categorised as mild when the total thickness was >5 but ≤8 mm, moderate when >8 but ≤11 mm, and marked when >11 mm. Osteoproliferative changes affecting the tympanic bullae were assessed by measuring the maximal bulla wall thickness. This was subjectively graded as mild when >4 but ≤6 mm, moderate when >6 but ≤8 mm, and marked when >8 mm. The range of normal tympanic bulla wall thickness reported in dogs based on CT measurements is between 0.6 and 3.9 mm (28). The location of any osteoproliferative changes affecting the calvarium was also recorded (i.e., frontal, parietal, occipital, or temporal bones). As for the assessment of the mandible, it was often impossible to differentiate the periosteal new bone from the underlying cortical bone of the calvarium. Therefore, the bone thickness (inner to outer edge of the calvarium) was recorded at the most affected site and subjectively graded as mild when >3 but ≤5 mm, moderate when >5 but ≤8 mm, and marked when >8 mm in total thickness. The lower limits for the osteoproliferative changes affecting the mandibles and calvarium were tentatively set by consensus of the authors, based on the maximum thickness of each bone in patients/areas that were unaffected (i.e., with no osteoproliferative changes). Where evidence of osteolysis was identified, it was characterised based on the typical patterns of bone lysis (permeative, moth-eaten, geographic), and subjectively graded as mild, moderate and marked.

**Figure 1:**
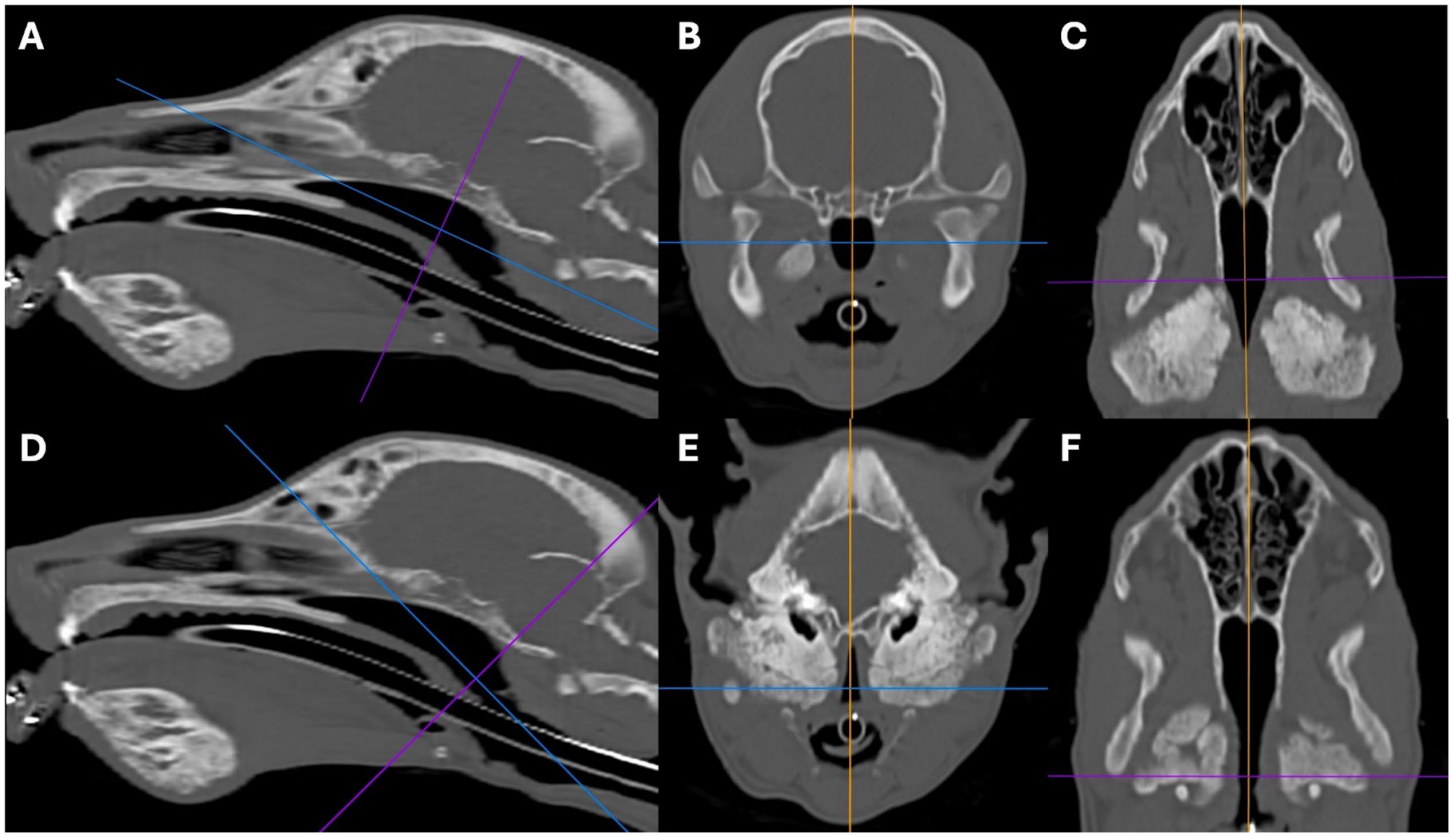
Sagittal **(A,D)**, transverse **(B,E)**, and dorsal oblique **(C,F)** multiplanar reconstruction images from a 5-month-old Scottish terrier showing how the severity of the nasopharyngeal narrowing was calculated. Images **A–C** show measurement of the maximum cross-sectional area of the nasopharynx, just caudal to the hamulus of the pterygoid bone. Images **D–F** show how the minimum cross-sectional area of the nasopharynx was measured.

The nasopharynx and external ear canals were evaluated for evidence of narrowing secondary to the osteoproliferation affecting the tympanic bullae and calvarium. The commonly identifiable regional lymph nodes (medial retropharyngeal and mandibular) were evaluated for evidence of enlargement. For each structure, findings were categorised as absent, mild, moderate, or marked. Nasopharyngeal narrowing was calculated using a multiplanar reconstruction (MPR). First, the maximum cross-sectional area (MaxCSA) of the nasopharynx was measured from a transverse plane image located immediately caudal to the hamulus of the pterygoid bone and oriented perpendicular to the soft palate (Figures 1A–C). Second, the minimum cross-sectional area (MinCSA) of the nasopharynx was measured from a transverse plane image at the site of maximal narrowing and oriented perpendicular to the soft palate (Figures 1D–F). The degree of nasopharyngeal narrowing was then calculated as a percentage of the maximal cross-sectional area: ((MaxCSA–MinCSA) / MaxCSA) x 100. External ear canal narrowing was calculated in a similar fashion. The maximum cross-sectional area of a grossly unaffected portion of the horizontal canal was measured, followed by the minimum cross-sectional area at the site of maximal narrowing. The degree of external ear canal narrowing was then calculated as a percentage of the maximal cross-sectional area. The degree of nasopharyngeal and external ear canal narrowing was subjectively categorised as mild when ≤25%, moderate when >25% but ≤50%, and marked when >50%. In patients without nasopharyngeal or external ear canal narrowing, the maximum and minimum cross-sectional area measurements (made at comparable sites to affected patients) were very similar to each other (i.e., producing a degree of narrowing close to 0%). A short-axis transverse diameter (SATD) of ≤8 mm was considered normal for the medial retropharyngeal and mandibular lymph nodes (29). The degree of lymph node enlargement was subjectively graded as mild when >8 but ≤12 mm, moderate when >12 but ≤16 mm, and marked when >16 mm.

### Statistics

Categorical data were summarised using frequency and percentages. Continuous data were summarised using the median and range. The severity of each of the six main CT features (mandibular osteoproliferation, tympanic bulla osteoproliferation, calvarial osteoproliferation, pharyngeal narrowing, external ear canal stenosis, and lymphadenopathy) was coded to a scale from absent (0), mild (1), moderate (2) to marked (3). The sum of these 6 scores was compared to patient age using Spearman rank correlation. A Mann–Whitney test adjusted for ties was used to compare combined severity in Scottish terrier with that of the remaining breeds. Data analysis was performed using Minitab 19; significance was set at *p*-value <0.05.

## Results

### Case information

A total of 20 dogs met the inclusion criteria. The clinical findings for each dog, as well as follow-up information (where available), are summarised in Table 1. The age at presentation varied from 6 weeks to 12 months old, with a mean age of 6.5 months. 11/20 patients (55%) were female, and 9/20 (45%) were male. 17/20 patients (85%) were entire, whilst 3/20 (15%) were neutered (2 male and 1 female). The most common breed within the patient cohort was the Scottish terrier (13/20; 65%). Three additional terrier breeds featured in the remaining dogs (one Cairn terrier, one Jack Russell terrier, and one Staffordshire bull terrier), along with four non-terrier breeds (one labrador retriever, one golden retriever, one akita, and one Slovakian rough-haired pointer). Overall, terrier breeds represented a total of 16/20 patients (80%) included in the study population.

**Table 1:** Patient information.

	Signalment	Centre	Presenting complaint	Physical examination	Presence of ankylosis	Treatment / outcome
Patient 1	8mo, FE, Scottish terrier	University of Pretoria	Pain when opening mouth/eating	Pain on mandibular palpation	No	NSAIDs + buprenorphine / None
Patient 2	4mo, ME, Scottish terrier	University of Pretoria	Pain when opening mouth/eating	Pain on mandibular palpation and enlarged LN	No	NSAIDs + buprenorphine / None
Patient 3	6mo, FE, Scottish terrier	University of Pretoria	Pain when opening mouth/eating, inability to open mouth more than 1cm	Enlarged LN	Yes	NSAIDs + tramadol/paracetamol / Caudal mandibulectomy. Temporary improvement of clinical signs. Euthanised. Post-mortem images of the skull included in Figure 7.
Patient 4	12mo, FE, Scottish terrier	University of Pretoria	Pain when opening mouth/eating, inability to open mouth	Enlarged LN	Yes	NSAIDs + buprenorphine / Bilateral TMJ osteotomy; post-op CT. Improved clinical signs. Bone histology.
Patient 5	8mo, FN, Scottish terrier	University of Pretoria	Pain when opening mouth/eating, inability to open mouth	Pain on mandibular palpation and enlarged LN	Yes	NSAIDs + tramadol/paracetamol / None
Patient 6	6mo, MC, Scottish terrier	University of Pretoria	Pain when opening mouth/eating	Pain on mandibular palpation and enlarged LN	No	NSAIDs + tramadol/paracetamol / Extraction of 102, 202 and 203 due to malocclusion
Patient 7	4mo, FE, Scottish terrier	University of Pretoria	Pain when opening mouth/eating	Pain on mandibular palpation and enlarged LN	Yes	NSAIDs + tramadol/paracetamol / None
Patient 8	12mo, FE, Scottish terrier	University of Pretoria	Pain when opening mouth/eating	Pain on mandibular palpation and enlarged LN	Yes	NSAIDs + methadone / Bilateral TMJ ostectomy
Patient 9	3mo, FE, Scottish terrier	University of Pretoria	Pain when opening mouth/eating	Enlarged LN	No	NSAIDs + tramadol/paracetamol / None
Patient 10	12mo, MC, Scottish terrier	University of Pretoria	Pain when opening mouth/eating, inability to open mouth	Enlarged LN	Yes	NSAIDs + methadone / Bilateral caudal mandibulectomy; condylectomy and coronoidectomy on the left.
Patient 11	10mo, ME, Scottish terrier	University of Pretoria	Pain when opening mouth/eating	Enlarged LN	No	NSAIDs + tramadol/paracetamol / None
Patient 12	5mo, ME, Scottish terrier	University of Pretoria	Pain when opening mouth/eating	Enlarged LN	No	NSAIDs + methadone / None
Patient 13	5mo, ME, Scottish terrier	University of Pretoria	Pain when opening mouth/eating	Pain on mandibular palpation and enlarged LN	No	NSAIDs + tramadol/paracetamol None
Patient 14	3mo, FE, Cairn terrier	Davies Veterinary Specialists	Pain when opening mouth/eating, PUO	Enlarged LN	No	NSAIDs + tramadol/paracetamol / None
Patient 15	3mo, FE, labrador	Davies Veterinary Specialists	Pain when opening mouth/eating, facial swelling	Unremarkable	No	NSAIDs + tramadol/paracetamol / None
Patient 16	5mo, FE, Slovakian rough haired pointer	Davies Veterinary Specialists	Pain when opening mouth/eating, PUO	Unremarkable	No	NSAIDs + tramadol/paracetamol / Ultrasound-guided FNAs of mandibular lymph nodes for cytology.
Patient 17	4mo, ME, Jack Russell terrier	Davies Veterinary Specialists	Pain when opening mouth/eating, facial/mandibular swelling	Unremarkable	No	NSAIDs + amoxicillin/clavulanic acid + tramadol/paracetamol / Mandible/bone histology and culture.
Patient 18	4mo, ME, Staffordshire bull terrier	Davies Veterinary Specialists	Pain when opening mouth/eating, facial swelling, PUO	Pain on mandibular palpation and enlarged LN	No	NSAIDs + paracetamol + gabapentin. Bone histology and culture / Resolved clinical signs (contacted after 3 months).
Patient 19	5mo, FE, golden retriever	Davies Veterinary Specialists	Pain when opening mouth/eating, PUO	Pain on mandibular palpation and enlarged LN	No	NSAIDs + tramadol/paracetamol / Resolved clinical signs in short term.
Patient 20	5mo, ME, akita	Davies Veterinary Specialists	Lethargy, hyporexia	Unremarkable	No	NSAIDs / Resolved clinical signs in short term

The most common presenting complaint in 19/20 patients (95%) was a variable degree of pain when opening the mouth, eating, or on palpation of the mandible. The most common physical examination finding was a variable degree of mandibular lymphadenopathy in 18/20 patients (90%), followed by: reduced ability or inability to open the mouth (secondary to TMJ pseudoankylosis or ankylosis) in 5/20 patients (25%); pyrexia in 4/20 patients (20%); and facial swelling in 3/20 patients (15%). The duration of clinical signs prior to referral ranged from 1 week up to 10 months; almost half of the patients 9/20 (45%) displayed clinical signs for 8–12 weeks prior to referral.

All dogs received one or more courses of pain relief prior to referral. Pain relief comprised various combinations of NSAIDs (meloxicam, robenacoxib), opiates (buprenorphine, tramadol, methadone), and acetaminophen with no improvement or mild improvement reported.

Surgery was performed in 5/20 patients (25%), all of which were cases from the University of Pretoria. One dog had multiple teeth extracted due to malocclusion and periodontitis. In the remaining four dogs, ostectomy of the excessive new bone formation surrounding the tympanic bullae and angular process of the mandible was performed, to alleviate the TMJ ankylosis. Additionally, one of these four dogs had a bilateral caudal mandibulectomy; the second had a bilateral mandibulectomy, with condylectomy and coronoidectomy performed on the left; the third underwent a bilateral TMJ ostectomy; and the first right upper molar tooth was extracted in the fourth. This fourth dog developed a fracture during the surgery, which required fixation, and subsequently developed periodontitis necessitating extraction of all teeth.

Bone biopsies were performed in 3/20 patients (15%), which were all submitted for histopathology and two were submitted for tissue culture. The culture was negative for both cases, whilst histopathology revealed evidence of bone remodelling, periosteal fibrosis, neutrophilic inflammation, and mild chronic haemorrhage. Ultrasound-guided fine needle aspirates (FNAs) of the mandibular lymph nodes were performed in a single patient (5%), which were submitted for culture and cytology. The culture was also negative for this case, whilst the cytology demonstrated evidence of neutrophilic and eosinophilic lymphadenitis.

### CT findings

**Figure 2:**
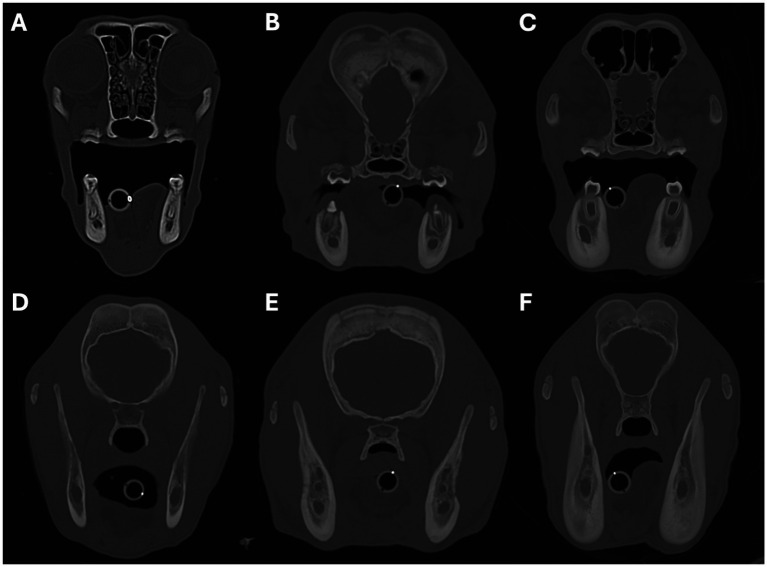
Transverse images showing examples of periosteal new bone formation affecting the mandibles. Images **A–C** show examples of osteoproliferation affecting the body of the mandible, and images **D–F** show osteoproliferation affecting the mandibular ramus. The severity of the osteoproliferation was graded as mild in images **(A,D)**, moderate in images **(B,E)**, and marked in images **(C,F)**. Image **A** is from a 5-month-old Scottish terrier, **B**,**E** are from a 4-month-old Jack Russell terrier, **C**,**F** are from a 5-month-old akita, and **D** is from a 5-month-old Slovakian rough-haired pointer.

**Figure 3:**
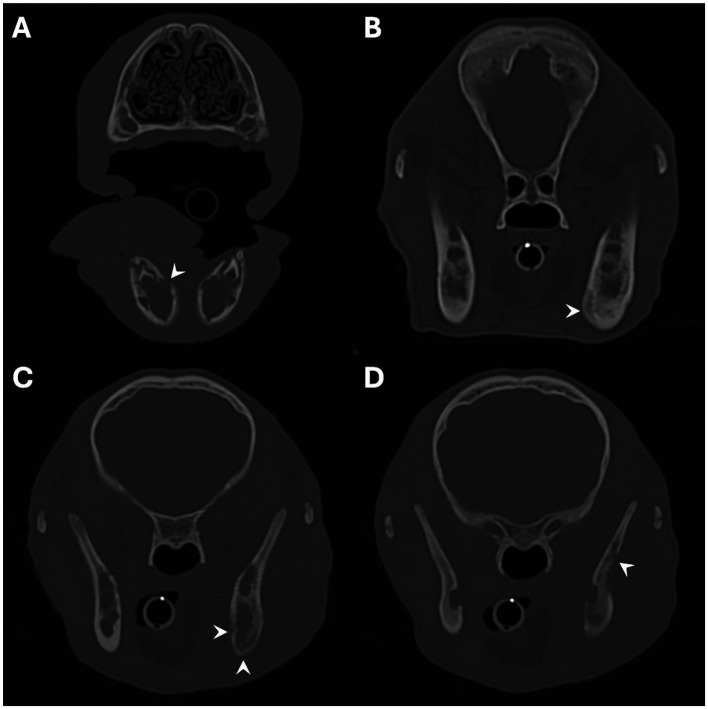
Transverse images showing examples of osteolysis affecting the mandibles. Images **A–C** show examples of osteolysis affecting the body of the mandible, and image **D** shows osteolysis affecting the mandibular ramus. The regions of osteolysis are denoted by the white arrowheads. Images **A**,**B** are from a 4-month-old Scottish terrier, and **C**,**D** are from a 4-month-old Jack Russell terrier.

CT findings for all 20 dogs are summarised in Table 2. All 20 patients included in the study showed variable amounts of periosteal new bone formation affecting the mandibles, which was categorised as mild in 1/20 (5%), moderate in 3/20 (15%), and marked in 16/20 (80%) dogs (Figure 2). The location of the mandibular new bone formation was also variable; the body was affected in 17/20 (85%), the ramus in 16/20 (80%), and the most rostral mandible in 5/20 (25%) dogs (Figure 2). 14/20 (70%) patients displayed new bone formation at more than one location on the mandible. When categorising the severity of the mandibular new bone formation for these patients, only the most severely affected site was recorded. The pattern of periosteal new bone formation was smooth for the body and rostral portion of the mandible, whilst palisading new bone formation was commonly observed on the mandibular ramus. In the 16/20 dogs with marked mandibular new bone formation, the underlying mandibular cortical bone could not be differentiated from the surrounding periosteal new bone. In two of the three dogs with moderate, and the single dog with mild mandibular new bone formation, the differentiation between the underlying mandibular cortical bone and the surrounding periosteal new bone formation was either faintly or clearly visible. A variable degree of sclerosis (defined as an increase in the attenuation of bone) was observed within the mandibular canal and surrounding the tooth roots in 18/20 (90%) patients. 10/20 patients (50%) demonstrated a degree of bone lysis, predominantly within the rostral mandible, which had an ill-defined and somewhat moth-eaten appearance (Figure 3). The osteoproliferative changes affecting the mandibles had a bilaterally symmetrical appearance in 17/20 (85%) patients. In 3/7 of the dogs where a post-contrast study was performed, a poorly contrast-enhancing hypo-attenuating rim was seen surrounding both mandibles.

**Table 2:** CT findings.

	Tympanic bullae						Mandibles				Cranium			Lymph nodes	
	Presence / grading of osteoproliferation	Uni / bilateral osteoproliferation	Osteolysis	TMJ involvement	Pharyngeal narrowing	Ear canal stenosis	Presence / grading of osteoproliferation	Location of osteoproliferation	Uni / bilateral osteoproliferation	Osteolysis	Presence / grading of osteoproliferation	Location of osteoproliferation	Osteolysis	Presence / grading of enlargement	Lymph node group
Patient 1	Marked	Bilateral	No	Yes	Moderate	No	Moderate	Body	Bilateral	No	No	–	No	Mild	MRP
Patient 2	No	–	No	No	No	No	Moderate	Body, ramus	Bilateral	No	Mild	Frontal	No	–	–
Patient 3	Marked	Bilateral	No	Yes	Marked	Moderate	Marked	Body, ramus, caudal portion	Bilateral	No	Moderate	Frontal, Parietal, Occipital	No	–	–
Patient 4	Marked	Bilateral	No	Yes	Moderate	Marked	Marked	Body, ramus	Bilateral	No	Marked	Frontal, Parietal	No	Mild	MRP
Patient 5	Marked	Bilateral	No	Yes	Marked	Marked	Marked	Body, ramus	Bilateral	No	Mild	Frontal	No	Mild	MRP
Patient 6	Marked	Bilateral	No	Yes	Marked	Moderate	Marked	Body, ramus	Bilateral	Yes	Mild	Frontal	No	Mild	MRP
Patient 7	No	–	No	No	No	No	Marked	Body	Bilateral	No	Moderate	Frontal, Parietal	No	Mild	MRP
Patient 8	Marked	Bilateral	No	Yes	Marked	Marked (complete)	Marked	Body, ramus	Bilateral	Yes	Moderate	Maxilla	No	Mild	MRP
Patient 9	Marked	Bilateral	No	Yes	Marked	Marked (complete)	Marked	Body, ramus	Bilateral	Yes	No	–	No	Moderate	MRP
Patient 10	Marked	Unilateral (left)	No	Yes	Marked	Marked	Marked	Body, ramus	Bilateral	No	Marked	Frontal, Parietal	No	Mild	MRP
Patient 11	Marked	Bilateral	No	Yes	Marked (nearly complete)	Moderate	Marked	Body, ramus	Bilateral	No	No	–	No	Mild	MRP
Patient 12	Marked	Bilateral	No	No	Marked (nearly complete)	Marked	Marked	Body, ramus	Unilateral (left)	Yes	Marked	Frontal, Parietal, Temporal	No	–	–
Patient 13	Marked	Unilateral (right)	No	Yes	Moderate	Mild	Marked	Body	Bilateral	Yes	Mild	Frontal, Parietal	Yes	Mild	MRP / Mandibular
Patient 14	Marked	Unilateral (left)	No	No	Moderate	Marked (complete)	Marked	Body, ramus	Bilateral	No	Marked	Frontal, Parietal, Occipital, Temporal	Yes	–	–
Patient 15	No	–	No	No	No	No	Marked	Body, ramus	Bilateral	Yes	Marked	Frontal, Parietal, Maxilla	Yes	Moderate	MRP / Mandibular
Patient 16	No	–	No	No	No	No	Mild	Ramus	Bilateral	Yes	Mild	Frontal, Parietal, Maxilla	No	Moderate	MRP / Mandibular
Patient 17	No	–	No	No	No	No	Moderate	Body, ramus	Bilateral	Yes	Mild	Frontal, Parietal, Occipital, Maxilla	No	–	–
Patient 18	No	–	No	No	No	No	Marked	Ramus	Bilateral	No	Marked	Frontal, Parietal, Occipital, Temporal, Maxilla	Yes	Mild	MRP
Patient 19	No	–	No	No	No	No	Marked	Ramus	Bilateral	Yes	Moderate	Frontal	Yes	Moderate	MRP / Mandibular
Patient 20	No	–	No	No	No	No	Marked	Body, ramus	Bilateral	Yes	Mild	Frontal, Parietal	No	Mild	MRP / Mandibular

**Figure 4:**
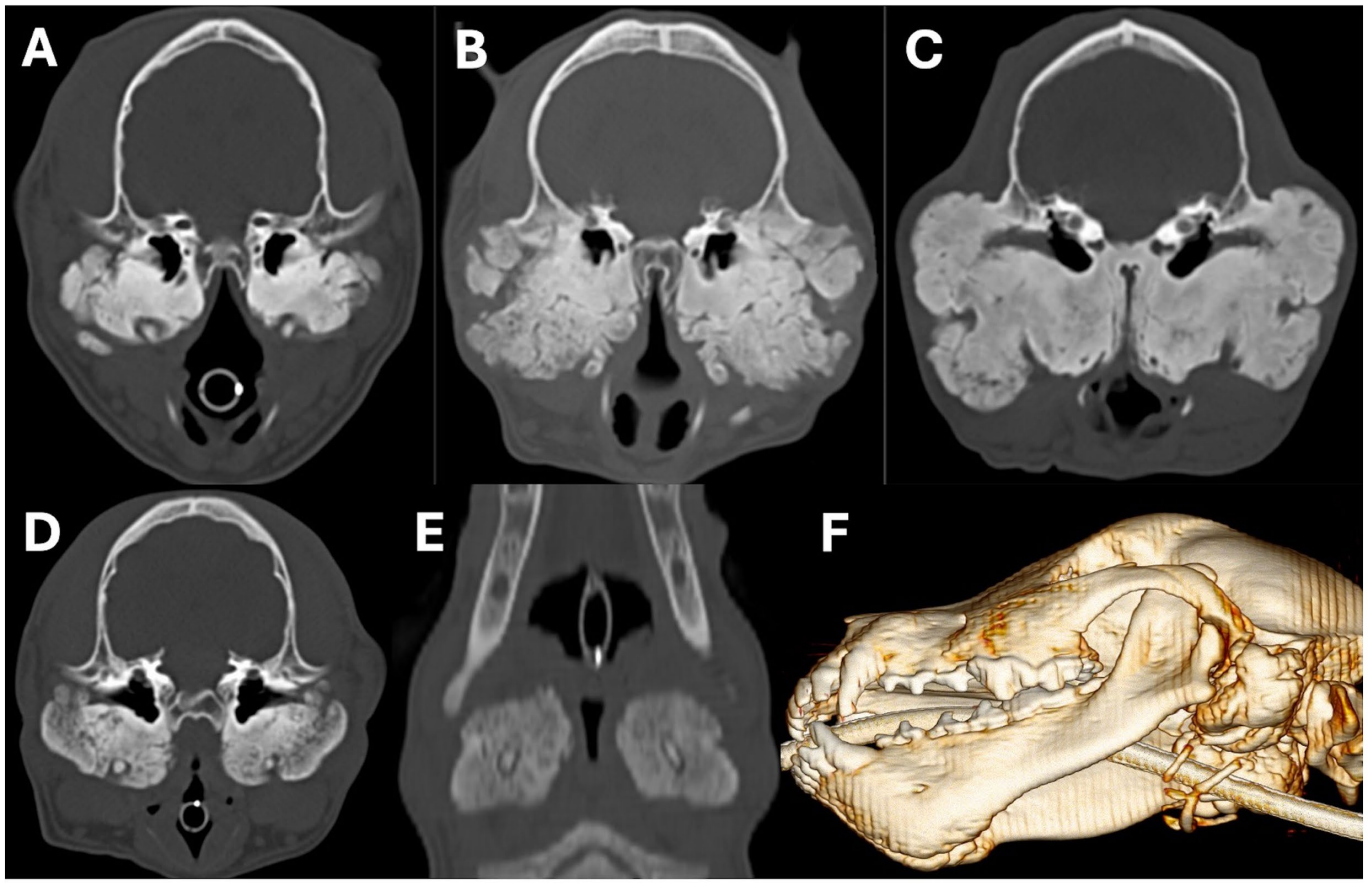
Transverse **(A–D)**, dorsal **(E)**, and sagittal oblique 3D volume rendered images showing examples of marked periosteal new bone formation affecting the tympanic bullae. In images **A**,**C**, the osteoproliferation has a predominantly solid and dense appearance; whereas in image **B**, it has an irregular lobular appearance. Images **D–F** show examples of the osteoproliferation surrounding the tympanic bullae extending around and engulfing the stylohyoid bones. Images **A**,**B** are from an 8-month-old Scottish terrier, **C** is from a 12-month-old Scottish terrier, and **D–F** are from a 6-month-old Scottish terrier.

Osteoproliferative changes affecting the tympanic bullae were observed in 12/20 (60%) patients. This ranged from areas of solid new bone formation to areas of lobulated new bone formation (Figures 4A–C). In all 12 patients with osteoproliferation, the severity of the periosteal new bone formation was graded as marked, and the osteoproliferative changes completely surrounded the bullae, extending along the ventral aspect of the horizontal ear canal, and surrounding the stylohyoid bones in 10 of these cases (Figures 4D–F). The underlying bone of the tympanic bulla wall was only faintly distinguished from the surrounding periosteal new bone formation in a single patient (8%). The changes were bilateral in 9/12 (75%) patients and unilateral in 3/12 (25%) patients (affecting the left bulla in two dogs and the right bulla in one dog). No evidence of osteolysis affecting the tympanic bullae was observed in any dog.

**Figure 5:**
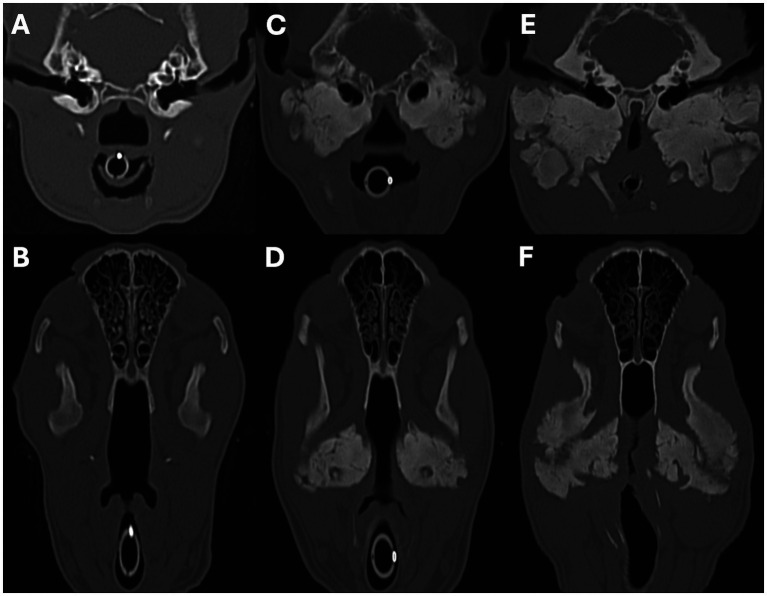
Transverse **(A,C,E)** and dorsal oblique **(B,D,F)** multiplanar reconstruction images showing examples of nasopharyngeal narrowing secondary to osteoproliferation surrounding the tympanic bullae. Images **A**,**B** are from a patient with no evidence of nasopharyngeal narrowing. Images **C**, **D** are from a patient with moderate nasopharyngeal narrowing, and images **E**,**F** are from a patient with marked nasopharyngeal narrowing. Images **A**, **B** are from a 4-month-old Scottish terrier, **C**,**D** are from a 6-month-old Scottish Terrier, and **E**,**F** are from an 8-month-old Scottish terrier.

**Figure 6:**
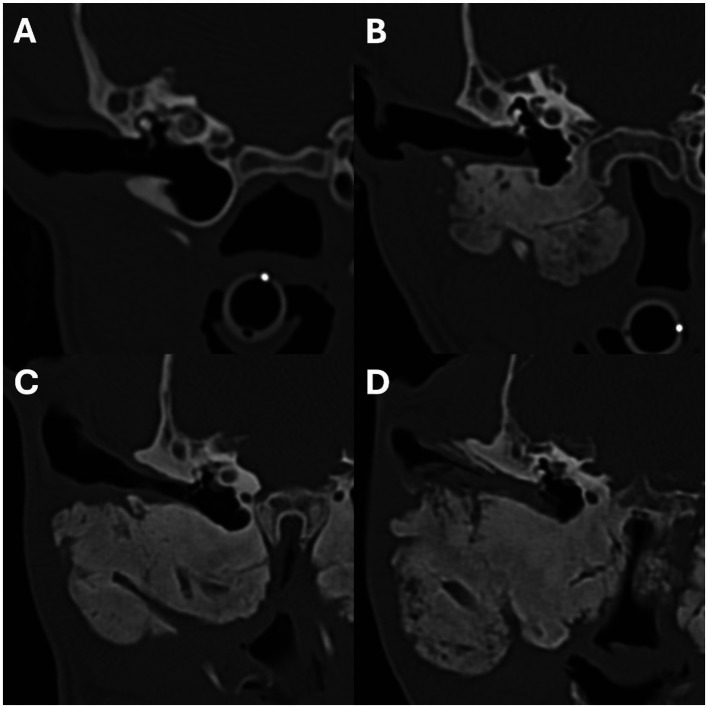
Transverse images showing examples of external ear canal stenosis secondary to osteoproliferation surrounding the tympanic bullae. Image **A** shows a normal horizontal ear canal with no evidence of stenosis. The remaining images show examples of patients with mild (image **B**), moderate (image **C**), and marked (imaged **D**) stenosis of the horizontal ear canal. Image **A** is from a 3-month-old labrador retriever, **B** is from a 5-month-old Scottish terrier, **C** is from an 8-month-old Scottish terrier, and **D** is from a 12-month-old Scottish terrier.

**Figure 7:**
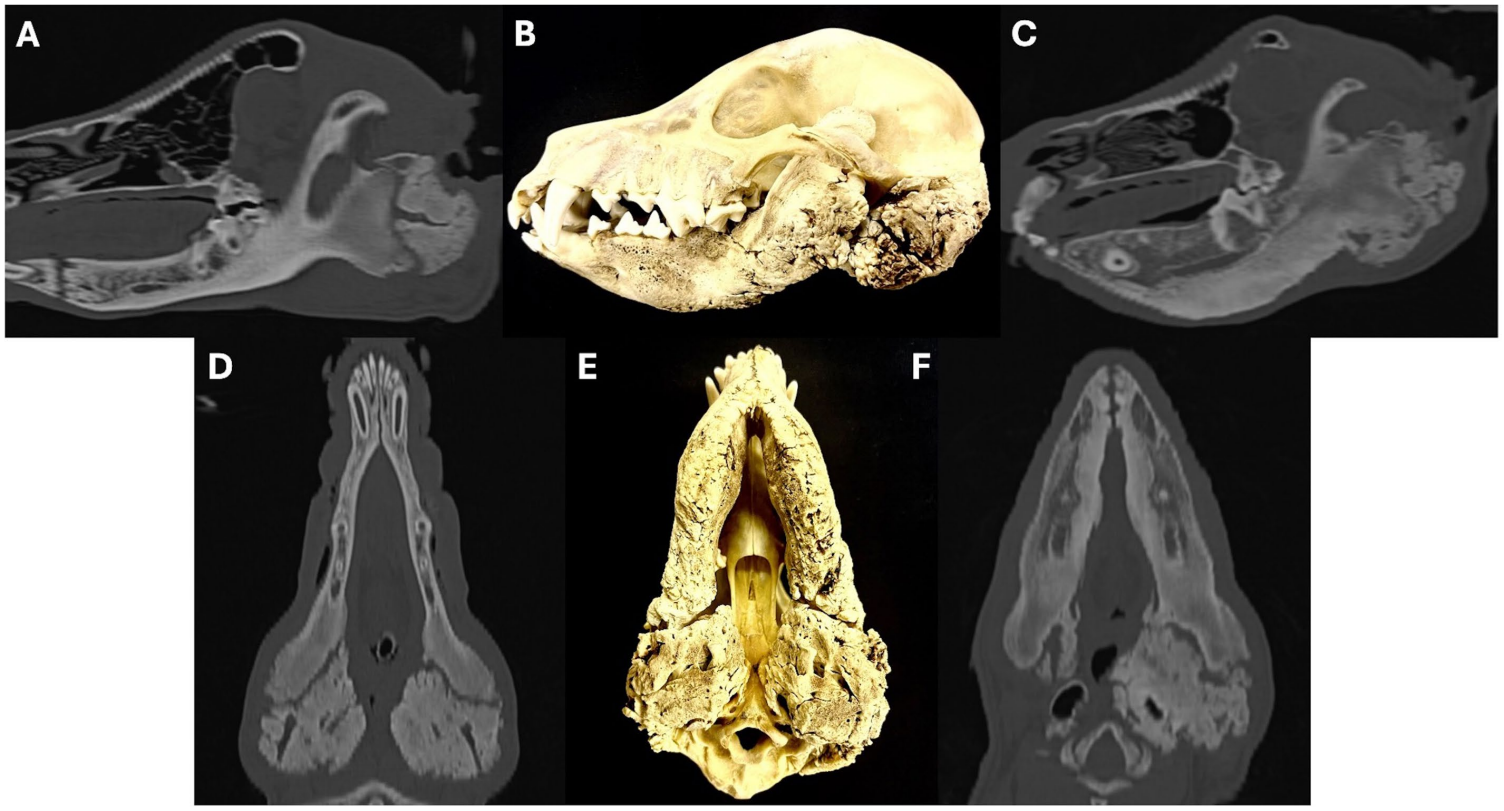
Sagittal oblique **(A,C)** and dorsal oblique **(D,F)** multiplanar reconstruction images showing examples of temporomandibular joint impingement secondary to osteoproliferation surrounding the tympanic bullae. Images **A**,**C** show how the proliferative new bone formation arising from the tympanic bulla extends rostrally and closely conforms to the caudal margin of the mandibular ramus, preventing the normal hinge-like opening of the temporomandibular joint. Images **D**,**F** show how the osteoproliferation also extends along the medial aspect of the mandibular ramus. Images **B**,**E** are photographs of a macerated skull showing severe osteoproliferation affecting the tympanic bullae and mandibular rami. Images **A**,**B**,**D**,**E** are from a 12-month-old Scottish terrier, **C**,**F** are from an 8-month-old Scottish terrier.

Narrowing or stenosis of the caudal nasopharynx was observed in all 12 patients (100%) that had osteoproliferative changes affecting the tympanic bullae. The degree of narrowing was graded as moderate in 4/12 (33%) patients and marked in 8/12 (67%; Figure 5). Near complete occlusion of the nasopharyngeal lumen was noted in 2/12 (16%) patients, in which the degree of narrowing was calculated as 90 and 98%. Horizontal ear canal narrowing, or stenosis, was present in 11/12 (92%) patients with osteoproliferative changes affecting the tympanic bullae and extending along the ventral aspect of the horizontal ear canal. The degree of horizontal canal narrowing was graded as mild in 1/11 (9%), moderate in 3/11 (27%) patients, marked in 4/11 (37%) patients, and there was complete occlusion of the lumen of the canal in 3/11 (27%) patients (Figure 6). The changes were bilateral in 8/11 (73%) and unilateral in 3/11 (27%) patients, left-sided in two of the dogs and right-sided in one of them. Restricted movement or impingement of the TMJ was suspected in 10/12 (83%) patients with marked osteoproliferative changes affecting the tympanic bullae. In these patients, the periosteal new bone formation arising from the tympanic bullae extended rostrally and closely conformed to the caudal margin of the mandibular ramus (between the condylar and angular processes) and also extended medial to the mandibular ramus (Figure 7). The TMJ impingement was bilateral in 8/10 (80%) patients and unilateral (left-sided) in 2/10 (20%) patients (Figure 7D). In one of the patients with unilateral TMJ impingement, there was a fusion of the periosteal new bone arising from the left tympanic bulla and the left mandibular ramus in some places (compatible with extra-articular ankylosis or pseudoankylosis).

**Figure 8:**
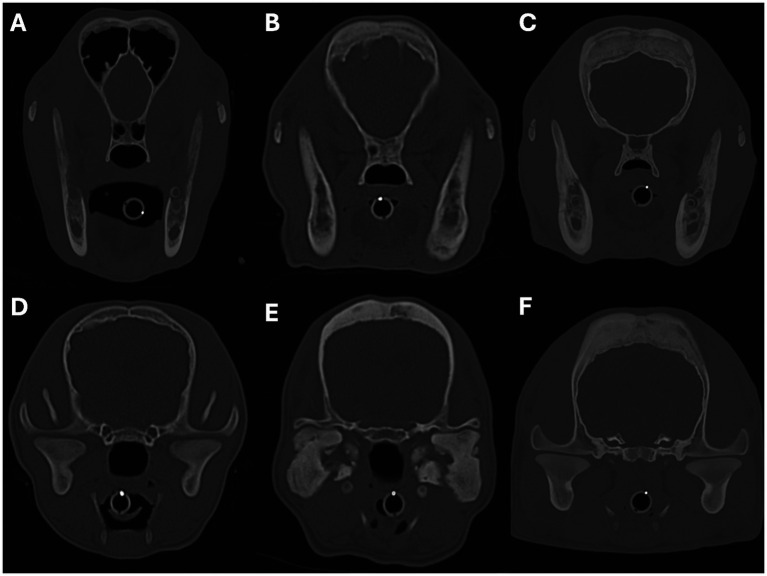
Transverse images showing examples of periosteal new bone formation affecting the cranium. Images **A–C** show examples of osteoproliferation affecting the frontal bones, and images **D–F** show osteoproliferation affecting the parietal bones. The severity of the osteoproliferation was graded as mild in images **A**,**D**, moderate in images **B**,**E**, and marked in images **C**,**F**. Image **A** is from a 4-month-old Slovakian rough-haired pointer, **B** is from a 4-month-old Cairn terrier, **C**,**F** are from a 4-month-old Staffordshire bull terrier, **D** is from a 4-month-old Jack Russell terrier, and **E** is from an 8-month-old Scottish terrier.

**Figure 9:**
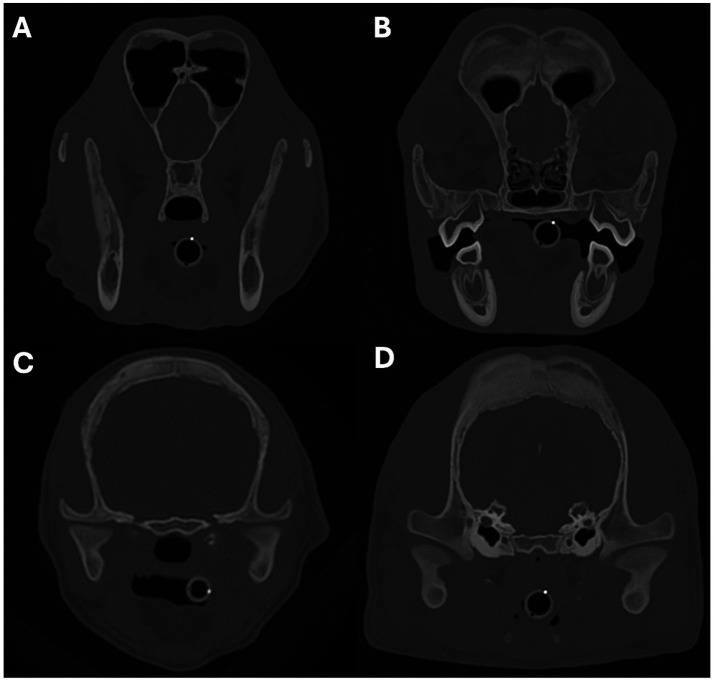
Transverse images showing examples of osteolysis affecting the cranium. Images **A**,**B** show examples of osteolysis affecting the frontal bones, and images **C**,**D** show osteolysis affecting the parietal bones. Image **A** is from a 5-month-old golden retriever, **B**,**D** are from a 4-month-old Staffordshire bull terrier, and **C** is from a 4-month-old Cairn terrier.

A total of 17/20 (85%) patients had osteoproliferative changes affecting the calvarium, with variable involvement of the frontal, parietal, temporal and occipital bones, or combinations of them (Figure 8). The proliferative new bone formation was graded as mild in 7/17 (41%) patients, moderate in 4/17 (24%) patients, and marked in 6/17 (35%) patients; the new bone formation could be differentiated from the underlying bone in those patients graded as mild and moderate, but not in those graded marked. Mild osteoproliferative changes affecting the maxilla were observed in 5/20 (25%) patients; in four of these five (80%), the underlying normal bone could be differentiated from the new bone formation. In 3/20 (15%) patients there was no evidence of any osteoproliferation affecting the cranium. There was evidence of calvarial osteolysis in 5/20 (25%) patients, predominantly affecting the frontal bone in four of them (80%) and the parietal bone in one of them (20%); the lysis was ill-defined and had a somewhat moth-eaten appearance (Figure 9). Only one patient (5%) showed mild osteolysis of the maxilla, which was bilateral and also moth-eaten in appearance.

A single patient showed proliferative new bone formation along the left caudal aspect of the spinous process of C2. The osteoproliferation alternated between formations of solid new bone and lobulated new bone. The dorsal arch of C1 also had a sclerotic appearance in 3/20 (15%) patients, although there was no clear evidence of osteoproliferative changes at this location.

Bilateral lymphadenomegaly of the regional lymph nodes (mandibular and/or medial retropharyngeal) was identified in 15/20 (75%) patients. Bilateral medial retropharyngeal lymphadenomegaly was observed in 15/20 patients (75%). Five of these patients 5/15 (33%) had concurrent mandibular lymphadenomegaly. The degree of lymphadenomegaly was graded as mild in 1/15 (7%) patients, moderate in 13/15 (86%) patients, and marked in 1/15 (7%) patients.

Considering the combination of CT features described above, Scottish terrier patients (65% of the total cohort) showed the most severe mandibular, tympanic bulla and calvarial osteoproliferation, as well as the most severe degree of external ear canal and nasopharyngeal narrowing.

**Figure 10:**
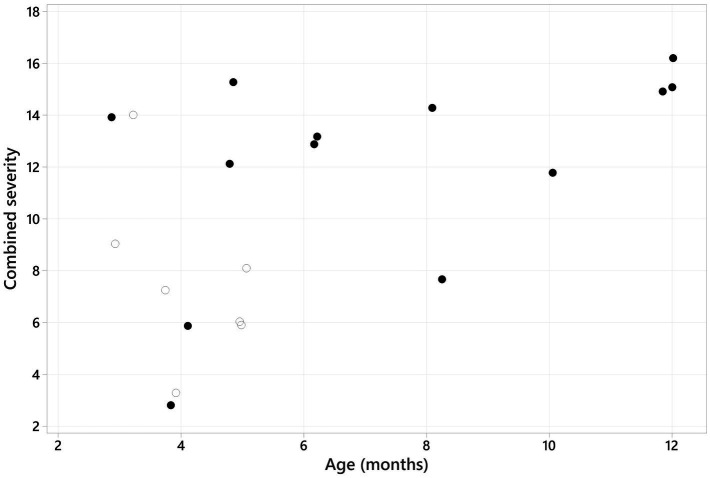
Scatter plot graph showing the combined severity (sum of the scores for each CT feature) plotted against the age of the patient. A positive Spearman rank correlation was identified (r_s_ = 0.481, *p* = 0.032). The solid black dots represent Scottish terriers, and the empty/white dots represent the remaining breeds.

**Figure 11:**
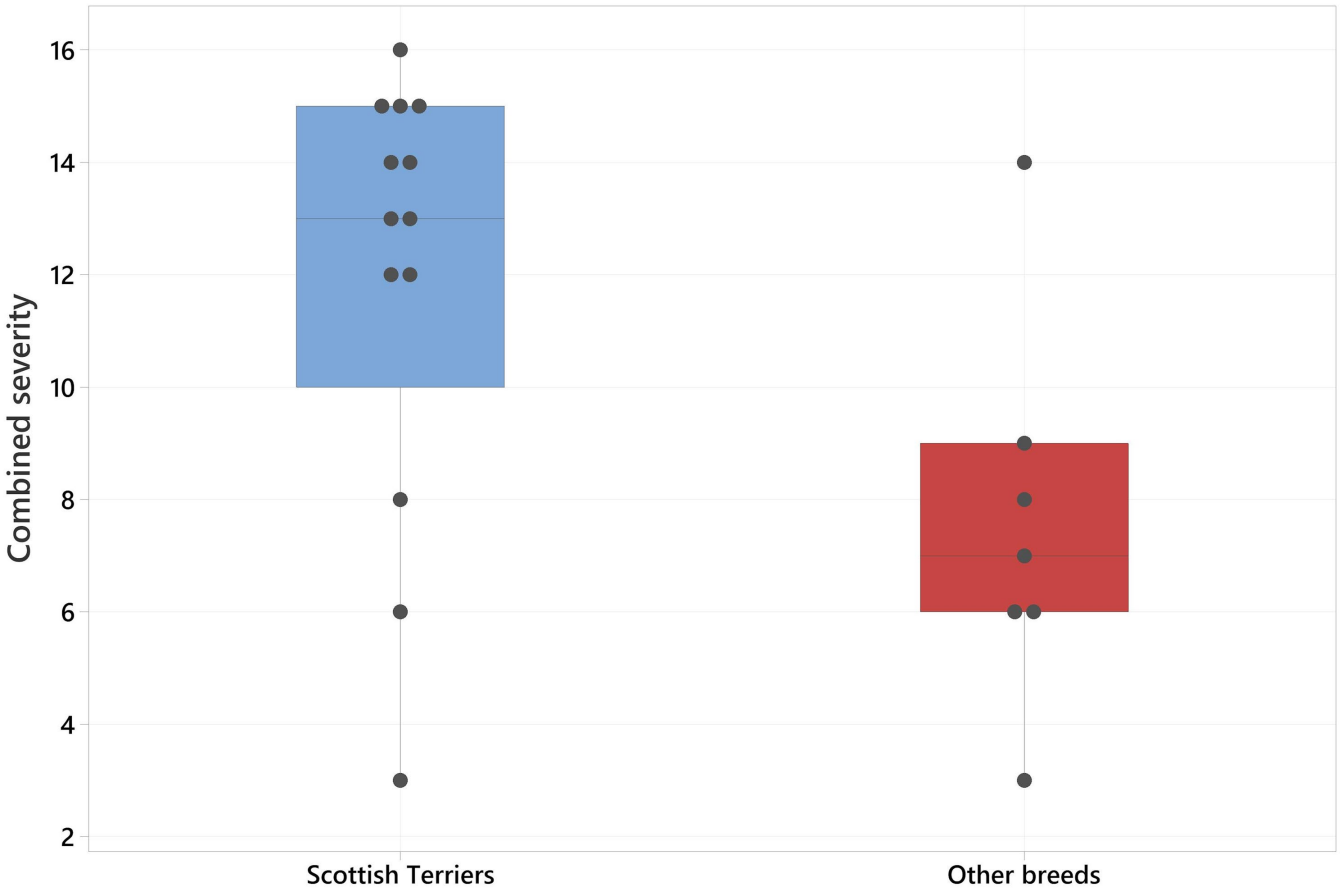
Boxplot showing the combined severity in Scottish terriers (blue box) versus the other breeds (red box). There was a significantly higher combined severity in Scottish terriers compared to other breeds (*p* = 0.038).

The Spearman correlation between the sum of the CT features of CMO and the age of the patient was significantly positive (r_s_ = 0.481, *p* = 0.032; Figure 10). The combined severity was significantly higher in Scottish Terriers compared to other breeds (medians of 13 and 7 respectively, *p* = 0.038; Figure 11).

## Discussion

This is the first published report focussed on describing the CT imaging features of CMO in a cohort of dogs. CT allows for detailed characterisation of the range of changes displayed in patients with CMO, which predominantly involve osteoproliferation affecting the mandibles, cranium, and tympanic bullae, and are largely in keeping with previous reports throughout the literature. However, the presence of osteolysis affecting the mandible and cranium was more prevalent among this study population than previously reported. Additionally, the features of TMJ impingement, external ear canal stenosis, nasopharyngeal narrowing and osteoproliferation surrounding the stylohyoid bone, which were all seen in dogs with marked osteoproliferation surrounding the tympanic bullae, have not previously been characterised in detail with any imaging modality.

Prior literature documenting the imaging features of CMO is largely focussed on the findings obtained from radiographs, which are well described. The most common features include bilaterally symmetrical proliferative new bone formation on the mandibles and tympanic bullae (2); however, osteoproliferation has also been identified on the bones of the cranium, the cervical vertebrae, and the radius and ulna (4, 17, 18). Descriptions of the CT features of CMO are limited to a handful of case reports (16–18, 23, 24), a case series including three WHWTs (21), and a study investigating the inheritance and genetic mapping of CMO in Deutsch drahthaar dogs where CT was used in 2/16 patients (1). As expected, the CMO features identified with CT in these cases closely resembled the known radiographic features. Periosteal new bone was identified on the mandible in all cases, whereas periosteal new bone was identified on the tympanic bullae in 6 cases (17, 18, 21, 24) and on the cranium in only 3 cases (17, 18, 23). There are only sporadic reports of the presence of sclerosis affecting the skull bones in cases of CMO that have been identified with radiography (14, 18, 23) or CT (16–18, 23). Even fewer descriptions exist for the presence of osteolysis affecting the skull bones in cases of CMO diagnosed with radiography (15) or CT (1). The presence of cortical lysis affecting the skull has been reported in a case report describing the MRI features of CMO (6).

In this study, the most common bony changes were osteoproliferation affecting the mandibles of all dogs (graded as marked in 80%, moderate in 15%, and mild in 5%), the cranium in 85% of dogs (graded as marked in 35%, moderate in 24%, and mild in 41%), and the tympanic bullae in 60% of dogs (all graded as marked). These findings are largely in keeping with those described in the previous literature; however, osteoproliferation was identified on the bones of the cranium much more frequently in this cohort of dogs. Whilst a reason for this difference remains unclear, the authors speculate that it could be attributed to the cross-sectional nature of CT imaging and the fact that the cranial changes were less severe than those affecting the mandibles and tympanic bullae (at least in our study population). The skull is a complex 3D structure making it challenging to assess on radiographs due to the superimposition of multiple bones on the 2D image (30). CT eliminates the problem of superimposition and allows the user to perform 3D multiplanar reconstructions (MPRs), which enables a fine-detail assessment of bony structures and the identification of much subtler changes. The features of the periosteal new bone formation identified at each site in this study are very similar to the nature of the osteoproliferation described for previous cases of CMO (2, 4, 17, 18). As in previous reports, the periosteal new bone formation affecting the cranium most often had a bilateral and largely symmetrical distribution. Bilateral changes were observed in the mandibles of 17/20 (85%) patients, the tympanic bullae of 9/12 (75%), and in all patients with cranial bone changes in this study. The aetiology of the hypo-attenuating and poorly contrast-enhancing rim surrounding the mandibles in three out of the seven patients that underwent a post-contrast series remains unclear as sampling, cytology, or histopathology were not performed on this tissue. The authors hypothesise that this could reflect oedema or a reactive inflammatory process within the soft tissues surrounding the mandibles.

Moderate or marked laterolateral narrowing of the caudal nasopharynx was observed in all 12 patients with marked osteoproliferative changes surrounding the tympanic bullae, which is attributed to ventromedial expansion of the new bone. The presence of nasopharyngeal compression associated with CMO has only been described once before in a dog with a severe and novel presentation of the disease with changes affecting the head, cervical vertebrae, and thoracic limbs identified with CT (17). None of the patients with evidence of nasopharyngeal narrowing, or indeed any patient in this study, had any history of or demonstrated clinical signs of dyspnoea or any other sign of upper respiratory tract dysfunction, which could be expected with compression or narrowing of the nasopharynx. Therefore, the presence of nasopharyngeal narrowing associated with osteoproliferative changes surrounding the tympanic bullae does not appear to be a clinically significant problem. One theory for this could be that as the new bone and nasopharyngeal narrowing develop gradually and progressively, it allows the patient time to adapt to them.

External ear canal stenosis was observed in 92% (11/12) of the patients with marked osteoproliferative changes surrounding the tympanic bullae, which was attributed to the extension of the new bone around the horizontal canal. The degree of stenosis was marked in the majority of affected patients in this study (8/11). The presence of ear disease associated with CMO has previously been described in four WHWTs. A case series described the surgical treatment of three WHWTs with chronic otitis media and externa, and para-aural abscessation secondary to CMO (21). These patients all had unilateral osteoproliferative changes affecting the left tympanic bulla and engulfing the ear canal, which the authors speculated likely predisposed to ear infection. Similarly, an additional case report describes the surgical and medical management of a WHWT with a drug-resistant chronic otitis media and externa, and para-aural abscessation secondary to CMO (24). Despite the presence of ear canal stenosis in more than half of the patients in this study, none of them had any clinical signs or prior history of ear disease.

Impingement or restriction in the range of motion of the TMJ was suspected in 83% (10/12) of patients with marked osteoproliferative changes affecting the tympanic bullae. However, on clinical examination, a suspicion of ankylosis / pseudoankylosis was identified in only five of these patients. The cases in which a restricted range of motion of the TMJ or pseudoankylosis was suspected from the CT images all demonstrated similar features. The marked osteoproliferation surrounding the tympanic bullae extended rostrally and conformed to the caudal margin of the mandibular ramus (between condylar and angular processes), and also extended along the medial aspect of the ramus. Marked bilateral mandibular osteoproliferation was also identified in all of these dogs. The combination of new bone formation at these two sites was suspected to cause an extra-articular or pseudoankylosis of the TMJ (31). Interestingly, one patient was reported as having ankylosis on clinical examination, which had normal tympanic bullae and no evidence of TMJ impingement on its CT study. The reason for this discrepancy between the suspicion of restricted TMJ motion identified at CT and the presence of ankylosis detected at clinical examination remains unclear. Possibilities for this could include concurrent undiagnosed masticatory muscle myositis, pain-related disuse atrophy, or secondary muscle fibrosis. The association between the osteoproliferative changes caused by CMO and restricted motion of the jaw/TMJ and ankylosis has long been known; it was reported in the early publications describing the features (5) and heritability (4) of the disease, as well as in a handful of cases within the last 10 years (9, 21). The reduced vertical range of motion of the mandible and the associated inability to open the mouth are the most important clinical consequences of CMO, alongside the presence of severe pain in some cases. Patients that are unable to open their mouths to eat will rapidly become malnourished (5). The only management options for such cases are alternative feeding modalities (oesophagostomy or percutaneous endoscopic gastrostomy tube feeding), salvage surgical procedures, or euthanasia.

Relatively mild bony changes (periosteal new bone formation or sclerosis) were identified in the cranial cervical vertebrae of four patients in this study. To the authors’ knowledge, abnormalities involving the cervical vertebrae have only been described in a case report of CMO in a mixed-breed dog with an unusual distribution of changes affecting the skull, cervical vertebrae, and thoracic limbs (17). However, the changes described in this case report were much more severe than the changes seen here. It is presumed that the mild cervical vertebral changes identified in this study were also an uncommon additional manifestation of CMO.

Osteolysis is rarely documented in association with CMO in the existing literature. A single case report documented osteolysis affecting the mandibles and cranium in a Shetland sheepdog (15). Osteolysis is not reported as a feature of CMO in any of the existing CT literature; however, in a paper that used CT in 2/16 Deutsch drahthaar dogs to confirm the diagnosis of CMO, the presence of mild osteolysis of the mandible can be seen in a single CT image from one of the dogs that is included as a figure within the manuscript (1). Nonetheless, the presence of osteolysis was not described within the manuscript itself. Osteolysis has also been reported in the mandibles and cranium of an Airedale terrier with CMO that underwent an MRI scan (6). Within this study’s cohort, mandibular osteolysis was identified in 10/20 dogs (50%), and calvarial osteolysis (predominantly affecting the frontal bone) was observed in 5/20 dogs (25%), with only 1/20 dogs (5%) showing lysis of the maxilla. In all cases, osteolysis affected the cortical bone and displayed an irregular moth-eaten pattern. The reason behind the increased prevalence of osteolysis in this group remains unclear; however, the capacity of CT to offer detailed assessments of bony structures and detect subtle changes beyond the reach of radiography is postulated. The detection of osteolysis with CT aligns with the recognised histopathological changes commonly associated with CMO, which are characterised by osteoclastic resorption of lamellar bone (5) and the presence of osteolysis in the periosteal or subperiosteal regions (10). Recognising osteolysis as a potential imaging hallmark of CMO holds clinical significance, as cortical bone lysis often suggests aggressive disease processes such as osteomyelitis or neoplasia. Notably, no evidence of infection or neoplasia was discerned via histopathology or tissue culture in the three dogs that underwent bone biopsy.

The age at presentation, reason for referral, and clinical signs within our study population closely mirrored those reported in the literature for dogs with CMO (1–3, 5, 14, 15, 21). Notably, a clear breed and geographic pattern were evident within our patient cohort, with all cases from the University of Pretoria (13/20) being Scottish terriers. These cases exhibited the most pronounced osteoproliferative changes, particularly affecting the tympanic bullae and mandibles, and showed the most severe signs of nasopharyngeal and external ear canal stenosis. Additionally, all patients with evidence of TMJ impingement were from the University of Pretoria (5/13), as were all of the patients that underwent surgery. The combined severity of the CT findings was found to be significantly higher in Scottish terriers compared to the remaining breeds (*p* = 0.038). Given this striking geographic dichotomy, there is a possibility that a genetic connection may exist among the South African cases through unknown common ancestors. Conversely, cases from Davies Veterinary Specialists comprised a mix of different breeds, and these patients demonstrated less severe osteoproliferative changes. It is also noteworthy that among the cases from the United Kingdom, four breeds were included (golden retriever, Jack Russell terrier, Slovakian rough-haired pointer, Staffordshire bull terrier) in which CMO has not previously been reported to the authors’ knowledge.

A significant positive correlation was found between the severity of the combined CT findings and the age of the patient (r_s_ = 0.481, *p* = 0.032). This is in keeping with the known self-limiting nature of CMO (1–3), whereby the osteoproliferative changes and bone remodelling are expected to cease once the animal reaches skeletal maturity. Furthermore, it is presumed that the severity of these changes would remain static in appearance once skeletal development ceases; however, the progression of the changes beyond skeletal maturity has not been investigated and remains unknown. The absence of long-term follow-up for the majority of patients in this study also prevents further assessment. Additional studies would therefore be required to better assess the progression of CMO changes following skeletal maturity and into adulthood.

This study has multiple limitations, several of which are inherently related to its retrospective nature. The CT images were acquired using different scanners at each hospital and the protocols used for image acquisition and subsequent image processing were not standardised, which resulted in variable image quality between cases. The absence of a post-contrast series in approximately two-thirds of cases (13/20) meant that assessment of any associated soft tissue changes in these patients was somewhat limited. The multicenter design of this study also contributed to variability in the information that was available in the medical records. Bone biopsies were only performed in 3/20 cases, meaning that histopathological confirmation of the diagnosis was not achieved in the majority of cases; however, it is common practice for the diagnosis of CMO to be based on appropriate signalment, history, clinical examination, and imaging findings. The small sample size is another limitation but is reflective of the uncommon nature of this disorder within the canine population. Finally, the absence of any WHWTs within this cohort of patients may represent a limiting factor when making comparisons with the available literature on CMO. The authors hypothesise this could be attributable to referring veterinarians being familiar with and used to diagnosing and managing CMO in WHWTs, meaning that such cases are less likely to be referred. Anecdotally, the authors also consider that WHWTs are not a particularly popular breed in South Africa.

In conclusion, CT enables detailed characterisation of the range of abnormalities displayed in dogs with CMO. The main findings closely resemble those reported throughout the existing literature and include proliferative new bone formation affecting the mandibles, cranium, and tympanic bullae. However, the prevalence of osteoproliferation affecting the cranium, and of osteolysis affecting the mandibles and cranium, was higher in this patient cohort compared to previous reports which likely reflects the ability of CT to detect much subtler bony changes compared with radiography. Additionally, this is the first study to provide a detailed description of the secondary effects that osteoproliferation has on the TMJ, ears, and nasopharynx. Whilst radiography is, and should remain, the first-line imaging modality to screen for and make a diagnosis of CMO, CT could prove useful for subtler cases where the diagnosis is not clear-cut, or to exclude other differential diagnoses or concurrent pathology. Furthermore, with the continued increase in the use of CT in veterinary practices and hospitals, it is helpful to document the range of features and accompanying changes that can be present with this disease. CT may also be a good choice for more severe cases of CMO where TMJ ankylosis is present and surgical options are being considered, as it provides a more detailed assessment of the distribution and extent of the new bone formation. It also enables multiplanar reformatting, generation of volume-rendered 3D images, and allows accurate measurements to be made, all of which can be useful for surgical planning purposes.

## Data Availability

The original contributions presented in the study are included in the article/supplementary material, further inquiries can be directed to the corresponding author.
